# Supplementary material for: Ticks and Chlamydia-Related Bacteria in Swiss Zoological Gardens Compared to in Contiguous and Distant Control Areas
Source: Microorganisms. 2023 Sep 30;11(10):2468. doi: 10.3390/microorganisms11102468 (PMC10609390; doi:10.3390/microorganisms11102468)

Figure S1: Weather according to the date of sampling. The red curve indicates the mean temperature. The bar plots indicate the rain. Wind speed and direction is represented with arrows. Under each picture is written the date. Next to each picture, you can see the corresponding samples. Above the red line of each picture is written the corresponding place: Forel and Mollie-Margot represent Servion region, le Vaud represent La Garenne region. Pictures were obtained using the Swiss weather mobile application.

20.07.22
Samples 1.1 to 1.8

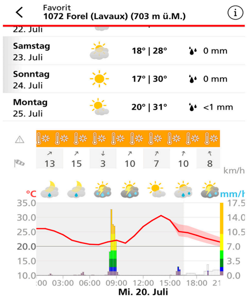

25.07.22
Samples 2.1 to 2.14

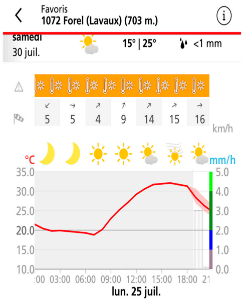

26.07.22
Samples 3.1 to 3.8

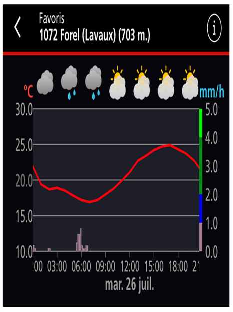

27.07.22
Samples 4.1 to 4.9

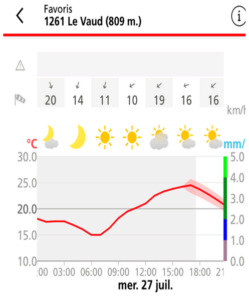

28.07.22
Samples 5.1 to 5.6

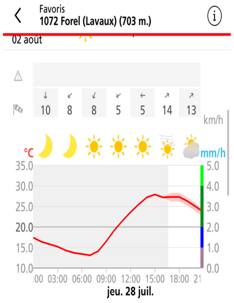

03.08.22
Samples 6.1 to 6.7

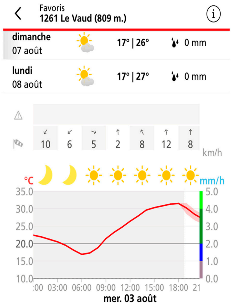

04.08.22
Samples 7.1 to 7.11

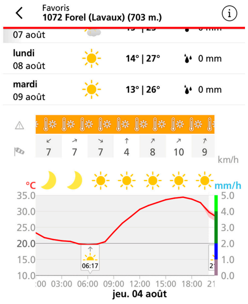

08.08.22
Samples 8.1 to 8.8

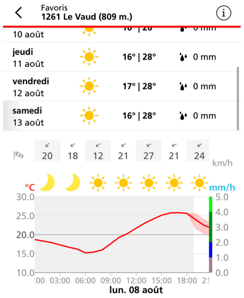

09.08.22
Samples 9.1 to 9.6

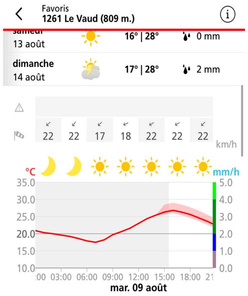

10.08.22
Samples 10.1 to 10.8

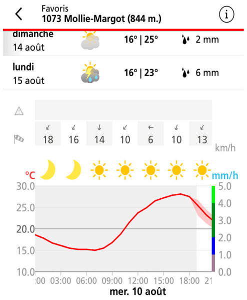

11.08.22
Samples 11.1 to 11.3

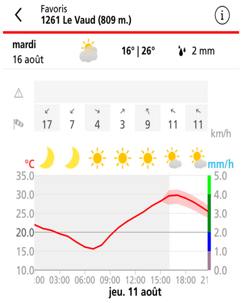

11.08.22
Samples 11.4 to 11.7

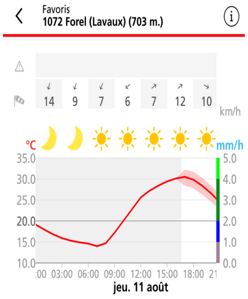

15.08.22
Samples 12.1 to 12.6

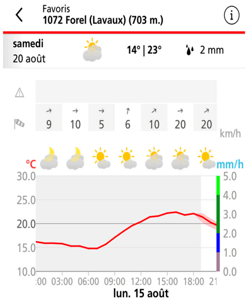

08.09.22
Samples 13.1 to 13.2

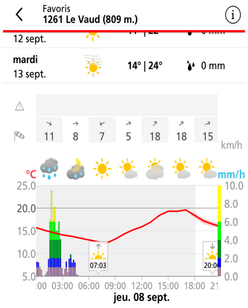

09.09.22
Samples 14.1 to 14.6

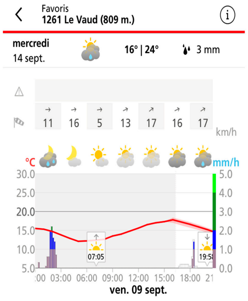

11.09.22
Samples 15.1 to 15.9

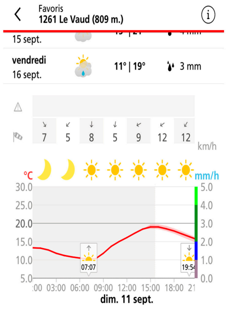

13.09.22
Samples 16.1 to 16.7

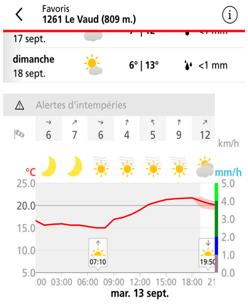

Supplement: Supplementary file 1 [file microorganisms-11-02468-s001.zip › Figure S1.pdf]
