# Supplementary material for: Ticks and Chlamydia-Related Bacteria in Swiss Zoological Gardens Compared to in Contiguous and Distant Control Areas
Source: Microorganisms. 2023 Sep 30;11(10):2468. doi: 10.3390/microorganisms11102468 (PMC10609390; doi:10.3390/microorganisms11102468)
Supplement: Supplementary file 1 [file microorganisms-11-02468-s001.zip › Figure S2.pdf]

Figure S2: Pictures from some of the flagging sessions. Enclosure of Bears, Alpacas, Wolfs, Snow Leopards, Goats - Vultures, and Badger are specified with icons. Surrounding enclosure of Parrots, Buffalos, Reindeers, Deers and Wild Pigs are specified with icons. A tick icon next to a picture indicates that at least 1 tick has been collected during the flagging session in which the picture has been taken.

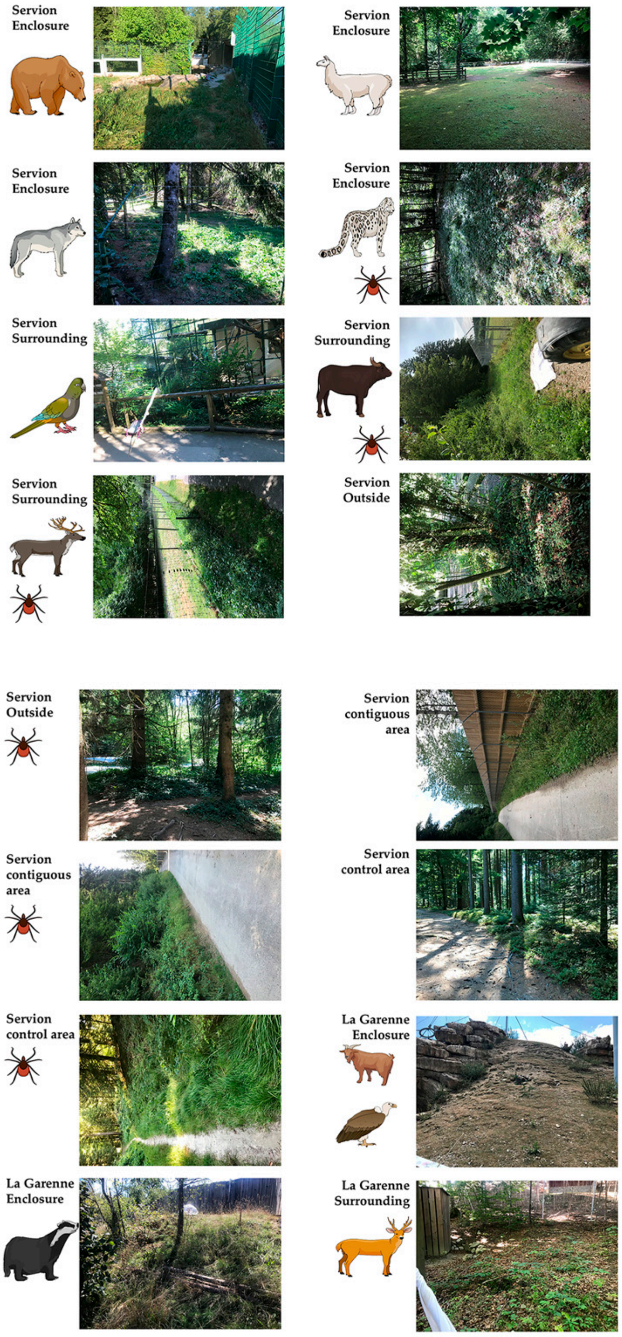

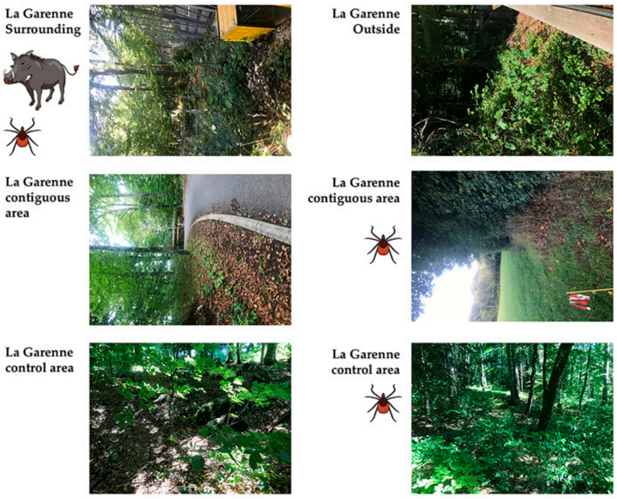
La Garenne Surrounding
La Garenne Outside
La Garenne contiguous area
La Garenne contiguous area
La Garenne control area
La Garenne control area

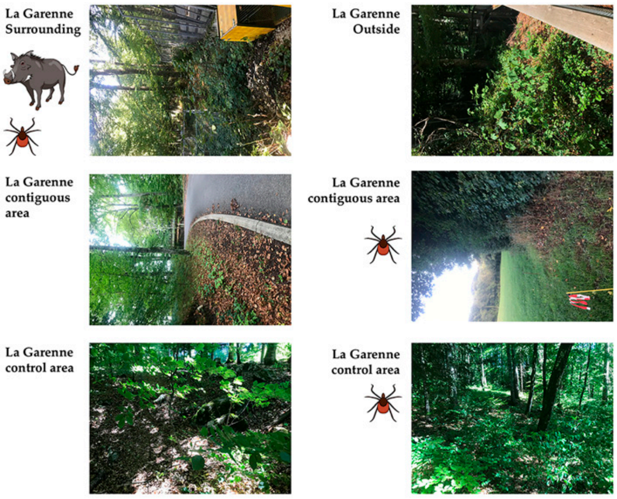
La Garenne Surrounding
La Garenne Outside
La Garenne contiguous area
La Garenne contiguous area
La Garenne control area
La Garenne control area
