# Supplementary material for: Ticks and Chlamydia-Related Bacteria in Swiss Zoological Gardens Compared to in Contiguous and Distant Control Areas
Source: Microorganisms. 2023 Sep 30;11(10):2468. doi: 10.3390/microorganisms11102468 (PMC10609390; doi:10.3390/microorganisms11102468)

Figure S3: Flag of 1m² used for the flagging sessions. Please note the white cotton shit, and the handle made of bamboo.

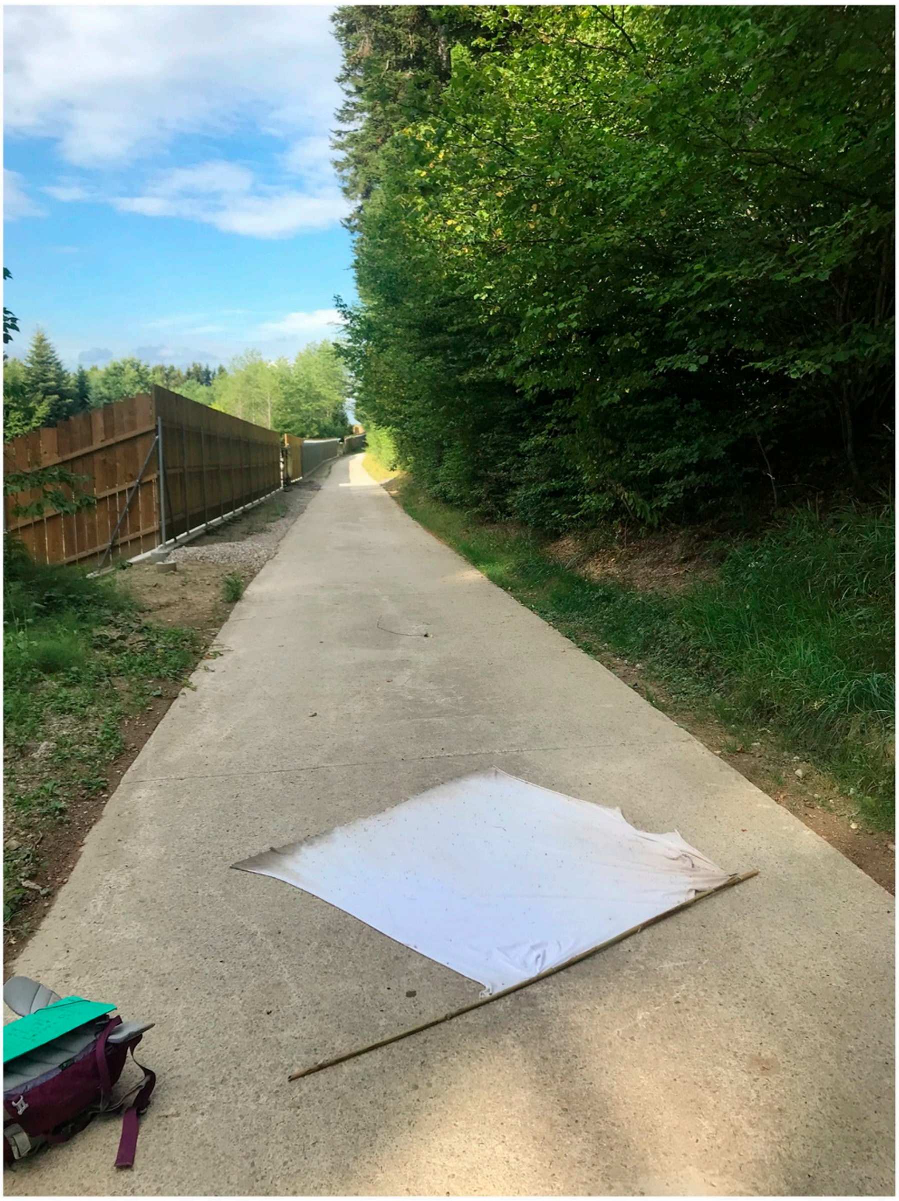

Supplement: Supplementary file 1 [file microorganisms-11-02468-s001.zip › Figure S3.pdf]
