# Supplementary material for: Ticks and Chlamydia-Related Bacteria in Swiss Zoological Gardens Compared to in Contiguous and Distant Control Areas
Source: Microorganisms. 2023 Sep 30;11(10):2468. doi: 10.3390/microorganisms11102468 (PMC10609390; doi:10.3390/microorganisms11102468)

Figure S4: Mean 16S rRNA gene copy number / µl according to the family of *Chlamydiae*. Please note the high mean 16S rRNA gene copy number / µl of *Rhabdochlamydiaceae*.

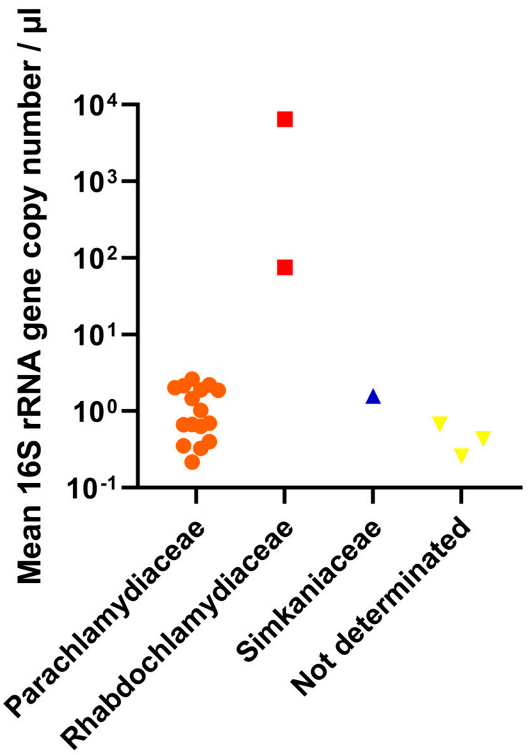

Supplement: Supplementary file 1 [file microorganisms-11-02468-s001.zip › Figure S4.pdf]
