# Supplementary material for: Ticks and Chlamydia-Related Bacteria in Swiss Zoological Gardens Compared to in Contiguous and Distant Control Areas
Source: Microorganisms. 2023 Sep 30;11(10):2468. doi: 10.3390/microorganisms11102468 (PMC10609390; doi:10.3390/microorganisms11102468)
Supplement: Supplementary file 1 [file microorganisms-11-02468-s001.zip › Table S2.pdf]

Table S2 : Total flagging time and Mean Flagging time of the different flagging areas. Mean flagging time for a place is obtained by dividing the total flagging time of that place with the number of sessions of that place, each session ranging from 2 to 10 minutes of flagging.

**Place**	**Total flagging time (min)**	**Mean Flagging time (min)**
La Garenne		
Zoo	194	9.4
-Enclosure	52	8.6
-Surrounding	70	10
-Outside	72	9
Contiguous area	260	9.6
Control area	84	9.3
**Servion**		
Zoo	198	8.4
-Enclosure	78	6
-Surrounding	40	8
-Outside	80	7.2
Contiguous area	218	9.4
Control area	130	10
