# Supplementary material for: Ticks and Chlamydia-Related Bacteria in Swiss Zoological Gardens Compared to in Contiguous and Distant Control Areas
Source: Microorganisms. 2023 Sep 30;11(10):2468. doi: 10.3390/microorganisms11102468 (PMC10609390; doi:10.3390/microorganisms11102468)
Supplement: Supplementary file 1 [file microorganisms-11-02468-s001.zip › Table S3.pdf]

Table S3: Mean ticks/min and Median ticks/min of different flagging areas. Mean ticks/min for a place represent the total of ticks/min of that place divided by the total amount of sessions at that place. Please note that combination of different places are represented in this table (for example Surrounding, Outside and contiguous area together).

Place	Mean (ticks/min)	Median (ticks/min)
La Garenne and Servion		
Enclosure	0	0
Surrounding, Outside	0.048	0
Surrounding, Outside, Contiguous area	0.112	0
Surrounding, Outside, Contiguous area, Control area	0.189	0
Contiguous area	0.152	0.1
Contiguous area, Control area	0.25	0.1
La Garenne		
Enclosure, Outside, Surrounding, Contiguous area	0.039	0
Enclosure, Outside, Surrounding, Contiguous area, Control area	0.121	0
Servion		
Enclosure, Outside, Surrounding, Contiguous area	0.137	0
Enclosure, Outside, Surrounding, Contiguous area, Control area	0.193	0
