# Supplementary material for: Ticks and Chlamydia-Related Bacteria in Swiss Zoological Gardens Compared to in Contiguous and Distant Control Areas
Source: Microorganisms. 2023 Sep 30;11(10):2468. doi: 10.3390/microorganisms11102468 (PMC10609390; doi:10.3390/microorganisms11102468)
Supplement: Supplementary file 1 [file microorganisms-11-02468-s001.zip › Table S5.pdf]

Table S5: Results of the basic local alignment search Tool (BLAST) analysis from the 16S rRNA gene sequences of the positive samples for pan-*Chlamydiae* TaqMan quantitative PCR (pC-qPCR). Percentage of similarity with specific species of *Chlamydiae* is shown in the third column. The 16S rRNA sequencing only allows identification to the Family-level lineage (second column). Note in the third column the accession numbers referring to specific species of *Chlamydiae,* and the Mean DNA/µl in the fifth column.

Sample°	Family-level lineage°°	Similarity (%)	Accession number (species)	Mean DNA/µl
2.9.6_1	*Parachlamydiaceae*	99	NR_025037	2,215
2.9.6_2	ND			
3.2.5_1	*Parachlamydiaceae*	97	NR_025037	1,869
3.2.5_2	*Parachlamydiaceae*	98	JN051144	1,869
3.3.2._1	*Parachlamydiaceae*	98	NR_025037	1,462
3.3.2_2	*Parachlamydiaceae*	99	JN051144	1,462
3.8.4_1	*Parachlamydiaceae*	98.5	NR_115817	2,143
3.8.4_2	*Parachlamydiaceae*	99	JN051144	2,143
5.1.1_1	*Parachlamydiaceae*	99	JN051144	2,632
5.1.1_2	*Simkaniaceae*	97	NR_074932	2,632
5.1.1_3	*Parachlamydiaceae*	100	not avalaible	-
5.1.1_4	*Parachlamydiaceae*	96	not avalaible	-
5.1.1_5	*Parachlamydiaceae*	98	not avalaible	-
7.1.5	*Parachlamydiaceae*	98	NR_115817	0.217
7.3.1_1	*Rhabdochlamydiaceae*	99	KF720713	75,205
7.3.1_2	*Rhabdochlamydiaceae*	99	KF720713	75,205
7.4.2	*Parachlamydiaceae*	96	JN051144	0,400
7.6.3_1	*Parachlamydiaceae*	99	JN051144	0,667
7.6.3_2	ND			0,667
7.7.1_1	*Simkaniaceae*	96	NR_074932	1,580
7.7.1_2	*Simkaniaceae*	96	NR_074932	1,580

8.4.2	*Parachlamydiaceae*	99	NR_115817	0,326
8.4.3	ND			0,257
8.6.2	*Parachlamydiaceae*	98	NR_026357	0,354
10.6.3_1<br>10.6.3_2	*Rhabdochlamydiaceae*<br>*Rhabdochlamydiaceae*	99<br>99	KF720713<br>KF720713	6471,756<br>6471,756
10.6.4	ND			0,431
11.3.4	*Parachlamydiaceae*	97	NR_025037	0,698
12.2.1	*Parachlamydiaceae*	98	NR_115817	0,671
12.5.5_1<br>12.5.5_2	*Parachlamydiaceae*<br>*Parachlamydiaceae*	99<br>98	JN051144<br>JN051144	2,015<br>2,015
12.5.9	ND			0,670
12.6.1	*Parachlamydiaceae*	98	AF083614	1,027
12.6.3	*Parachlamydiaceae*	97	JN051144	0,631
13.2.1	*Parachlamydiaceae*	98	JN112799	1,892

° The first number indicates the day of the sessions, the second indicates the session of that day, the third the number of the tick, and the 4th number which duplicate it is. For example, the tick called 2.9.6_1 was collected during the second day of flagging, it was the 9th session of that day, and was the 6th tick of that session, 1st of the duplicate.
°° ND = not determinated.
